# Time series analysis reveals synchrony and asynchrony between conflict management effort and increasing large grazing bird populations in northern Europe

**DOI:** 10.1111/conl.12450

**Published:** 2018-03-25

**Authors:** Jeremy J. Cusack, A. Brad Duthie, O. Sarobidy Rakotonarivo, Rocío A. Pozo, Tom H.E. Mason, Johan Månsson, Lovisa Nilsson, Ingunn M. Tombre, Einar Eythórsson, Jesper Madsen, Ayesha Tulloch, Richard D. Hearn, Steve Redpath, Nils Bunnefeld

**Affiliations:** ^1^ Biological and Environmental Sciences University of Stirling Stirling FK9 4LA United Kingdom; ^2^ Conservation Ecology Group, Department of Biosciences Durham University United Kingdom; ^3^ Wildlife Damage Center, Grimsö Research Station, Department of Ecology Swedish University of Agricultural Sciences Sweden; ^4^ Department of Arctic Ecology Norwegian Institute for Nature Research Norway; ^5^ High North Department Norwegian Institute for Cultural Heritage Research Tromsø Norway; ^6^ Department of Bioscience Aarhus University Denmark; ^7^ Centre for Biodiversity and Conservation Science, School of Earth and Environmental Sciences University of Queensland Brisbane Australia; ^8^ Wildfowl & Wetlands Trust Slimbridge Gloucestershire GL2 7BT United Kingdom; ^9^ Institute of Biological and Environmental Sciences University of Aberdeen United Kingdom; ^10^ Grimsö Research Station, Department of Ecology Swedish University of Agricultural Sciences Sweden

**Keywords:** compensation, conflict, crane, goose, harvest, management, population count, scaring, time series

## Abstract

The management of conflicts between wildlife conservation and agricultural practices often involves the implementation of strategies aimed at reducing the cost of wildlife impacts on crops. Vital to the success of these strategies is the perception that changes in management efforts are synchronized relative to changes in impact levels, yet this expectation is never evaluated. We assess the level of synchrony between time series of population counts and management effort in the context of conflicts between agriculture and five populations of large grazing birds in northern Europe. We reveal inconsistent patterns of synchrony and asynchrony between changes in population counts and impact management effort relating to population harvesting, monetary payments, or scaring practices. This variation is likely due to differing management aims, the existence of lags between management decisions and population monitoring, and the inconsistent use of predictive models across case studies. Overall, our findings highlight the need for more adaptive and timely responses of management to changes in target species numbers so as not to unexpectedly increase social conflicts and jeopardize the status of wildlife populations.

## 1 INTRODUCTION

**Figure 1:**
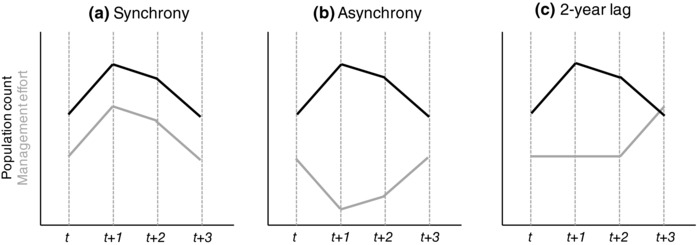
Schematic representations of synchrony (a), asynchrony (b), and a 2‐year time lag (c) in management effort relative to population count

Conflicts between wildlife conservation and agricultural activities occur worldwide and have serious consequences for biodiversity and human well‐being (Barua, Bhagwat, & Jadhav, 2013; Hill, 2015). These conflicts often emerge due to the impacts that wild animals have on agricultural practices and production, such as crop damage, and the management strategies put in place to reduce these impacts (Redpath et al., 2013; Young et al., 2010). Strategies include population control through harvesting or culling (Treves & Naughton‐Treves, 2005), compensation or subsidy schemes (Nyhus, Osofsky, Ferraro, Madden, & Fischer, 2005; Schwerdtner & Gruber, 2007), and mitigation techniques such as nonlethal scaring or barriers (Simonsen, Madsen, Tombre, & Nabe‐Nielsen, 2016; Sitati & Walpole, 2006). A key requirement to the success of these strategies in attenuating conflicts is that management efforts are adapted to current wildlife impact levels (Majić, de Bodonia, Huber, & Bunnefeld, 2011; Reiter, Brunson, & Schmidt, 1999). In other words, stakeholders in a conflict expect managers to make decisions that match changes in management effort (e.g., culling quotas or monetary compensation) to changes in wildlife impact levels, thereby resulting in a degree of synchrony between the two (Figure 1a), yet this assumption is rarely evaluated.

The paucity of studies examining the relationship between changes in management and wildlife impact levels is surprising for several reasons. First, the management of wildlife populations is increasingly described as adaptive, which by definition implies a response rooted in available scientific monitoring (Bunnefeld, Hoshino, & Milner‐Gulland, 2011). Hence, it can be expected that the level of management effort will be dependent on measured indicators of wildlife impacts, such as species population size or distribution (Simonsen, Tombre, & Madsen, 2017). Second, delays or deficiencies in the implementation of management actions may strengthen attitudes against wildlife conservation (Webber, Hill, & Reynolds, 2007), thus exacerbating the conflict (Olson et al., 2015). This can be expected if population culling quotas or compensation payments are reduced from one year to the next despite a measured or perceived increase in the abundance of a target species (i.e., asynchrony; Figure 1b). Last, delayed management actions may be ill suited to the ecological context in which they are eventually applied, thereby resulting in overabundance or extinction risks (Fryxell, Packer, McCann, Solberg, & Sæther, 2010; Figure 1c).

In this study, we assess the level of synchrony between historical time series of population counts and management effort spanning 9 to 24 years collected in the context of conflicts between agriculture and the conservation of five populations of large grazing birds (geese and cranes) during their wintering and staging periods in northern Europe. Many populations of large grazing birds have undergone exponential growth and expanding distributions in recent decades owing to an increase in protective legislation, improvements in agricultural land quality, and global climate change (Fox, Elmberg, Tombre, & Hessel, 2017; Mason, Keane, Redpath, & Bunnefeld, 2017). Although this has significantly improved the conservation status of many species (Fox & Madsen, 2017; Harris & Mirande, 2013), it has also led to widespread conflict with farmers, and to increased calls for impact management by members of the agricultural community (see Fox et al., 2017 and references therein for a review of crop losses caused by herbivorous waterfowl). As an example, it is estimated that on the Scottish island of Islay the cost of conflict between farmers and goose conservation is in excess of €1 m (McKenzie & Shaw, 2017). As a result, a number of management schemes have been developed to reduce the costs of agricultural damage, all the while maintaining populations at a favorable conservation status. In this context, we analyze historical time series collected from a subset of these schemes, aiming to evaluate the level of synchrony between population counts and relevant harvesting, monetary payment, and scaring effort levels.

## 2 METHODS

### 2.1 Study sites and species

**Figure 2:**
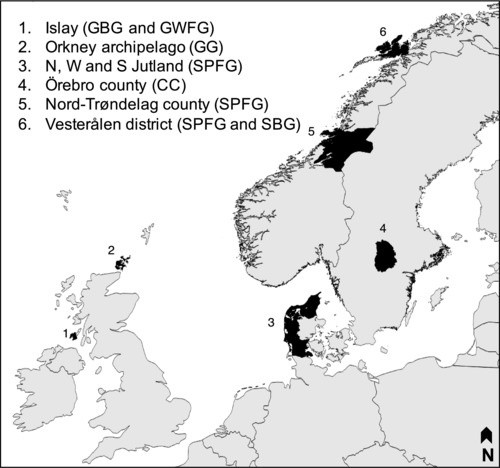
Location of case study sites across northern Europe. Acronyms between parentheses in the legend refer to species populations, namely Svalbard pink‐footed and barnacle geese (SPFG and SBG, respectively), Greenland white‐fronted and barnacle geese (GWFG and GBG, respectively), greylag geese (GG), and common cranes (CC)

We considered time series of population counts and management effort collected for six case studies in northern Europe (Figure 2), and involving five populations of four species of goose (Greenland and Svalbard barnacle geese *Branta leucopsis*, Greenland white‐fronted goose *Anser albifrons flavirostris*, greylag goose *Anser anser* and Svalbard pink‐footed goose *Anser brachyrhynchus*) and one species of crane (common crane *Grus grus*), all of which are known to cause damage to agricultural crops. Case studies included two island archipelagos (the isle of Islay and the Orkney archipelago in the United Kingdom), one Norwegian county (Nord‐Trøndelag in central Norway), one Norwegian district (Vesterålen in northern Norway), one Swedish county (Örebro in south‐central Sweden, including Kvismaren Nature Reserve where >90% of staging cranes are found), and the northern, western, and southern regions of the Jutland peninsula in Denmark (hereafter, Jutland). Case study area ranged from 620 km^2^ for Islay to 22,412 km^2^ for Nord‐Trøndelag, with a total area considered of 57,458 km^2^.

### 2.2 Time series data

For each case study, we collated time series of annual counts for the target species and relevant management effort (Table 1, but see also Supporting Information S1 for additional details). When multiple surveys were performed within a given year, we averaged the resulting counts and related this to management effort. However, when estimating count trends for a given species (see below), all counts were included so as to obtain a more precise trend estimate.

**Table 1:** Summary of annual count and management effort time series collected for each case study

				Count		Management effort		
Country	Site	Administrative level	Species	Time series	Span	Time series	Span	References
Denmark	Jutland	Region	Svalbard pink‐footed goose	Flyway count carried out in November	1991 – 2015	Reported winter harvest	1991 – 2015	Madsen et al., 2015; Clausen, Christensen, Gundersen, & Madsen, 2017
Norway	Nord‐Trøndelag	County	Svalbard pink‐footed goose	Flyway count carried out in November	1991 – 2015	Reported winter harvest	1991 – 2015	Clausen et al., 2017, Madsen et al., 2015
Norway	Vesterålen	District	Svalbard pink‐footed goose Svalbard barnacle goose	Average number of geese counted across four municipalities^a^ between 7^th^ and 20^th^ May	1987 – 2015	Average subsidy per application from four municipalities^a^	2007 – 2016	Tombre et al., 2013
Sweden	Örebro	County	Common crane	Maximum count recorded across county staging sites between March and October	1990 – 2015	Average compensation per damage report between March and October Total funding allocated to nonlethal scaring activities	2003 – 2015	Nilsson, 2016; Nilsson, Bunnefeld, Persson, & Månsson, 2016
UK	Islay	Island	Greenland barnacle goose Greenland white‐fronted goose	Winter month counts (Nov to April)	1987 – 2015	Winter harvest^b^ Average compensation payment per management scheme participant Total funding allocated to nonlethal and lethal scaring activities	2000 – 2015	McKenzie and Shaw, 2017 (and references therein)
UK	Orkney	Archipelago	Resident (British) and migrant (Icelandic) greylag goose	Winter population count (both populations combined)	1982 – 2015	Total number of shooting licenses issued (both populations combined)	2006 – 2016	Churchill & Skene, 2016

a The four municipalities were Sortland, Andøy, Hadsel, and Øksnes. The annual population count refers to the total number of geese counted across these four municipalities between 7th and 20th May divided by the number of survey days (Tombre et al., 2013).

b Only for Greenland barnacle geese.

Three main types of management actions were considered in this study: harvesting, monetary payments, or scaring. Harvesting referred to the shooting of a set number of individuals during a given period (e.g., open season), be it for sport, culling, or lethal scaring purposes. It was recorded as the winter hunting bag (i.e., total number of individuals harvested over a set number of winter months) in the case of the Svalbard pink‐footed goose in Jutland (open season from 1st September to 31st December) and Nord‐Trøndelag (10th August to 23rd December), as well as for the Greenland barnacle goose on Islay (October and March), and as the number of licenses issued to hunt resident British and migratory Icelandic greylag geese in Orkney (October to January). Monetary payments included: (1) financial compensation made to cover the cost of damage to crops after it had occurred (in Örebro), and (2) subsidies paid in advance to cover the cost of expected damage or the setting aside of grazing areas (on Islay and in Vesterålen). Last, scaring consisted of practices aimed at preventing flocks of geese or cranes from damaging unharvested crops. Although a diverse range of scaring practices is likely implemented on an ad hoc basis at many of the sites considered, effort in each case has not been quantified. We therefore used expenses relating to scaring activities as an indicator of scaring effort in Örebro and on Islay. All time series data are supplied in the Supplementary Information S2.

### 2.3 Long‐term synchrony

For each case study, we modeled separate trends for counts and management effort over time using generalized additive models (GAMs; see Supporting Information S3). In all cases, time series were of yearly frequency and continuous (i.e., no missing years). We assessed trend synchrony by carrying out binomial tests on the proportion of time steps for which the estimated management and count trends behaved in the same way (i.e., both significantly increasing/decreasing, and showing no significant trend). As we expected a random management effort time series to be stationary (i.e., showing no trend on average), the expected probability in the binomial test was taken as the proportion of time steps that showed neither a significant increasing nor decreasing count trend.

### 2.4 Short‐term synchrony

We applied a measure of synchrony (*φ*) combined with a randomization procedure that shuffled values within each time series independently to test whether annual changes in count and management effort were more synchronous than expected by chance in the short term (see Supporting Information S4). Using the *community.sync* function in the R package synchrony (Gouhier & Guichard, 2014), we obtained an expected distribution for *φ* from 1,000 iterations and carried out a two‐tailed test to infer whether the observed value of synchrony was greater or lower than expected by chance. Using the same approach, we also tested the level of synchrony between management effort at time *t* and population count at times *t* ‐ *l*, where *l* represented a time lag of 1, 2, or 3 years. All analyses were performed in R version 3.3.3 (R Core Team, 2017).

## 3 RESULTS

### 3.1 Long‐term synchrony

**Figure 3:**
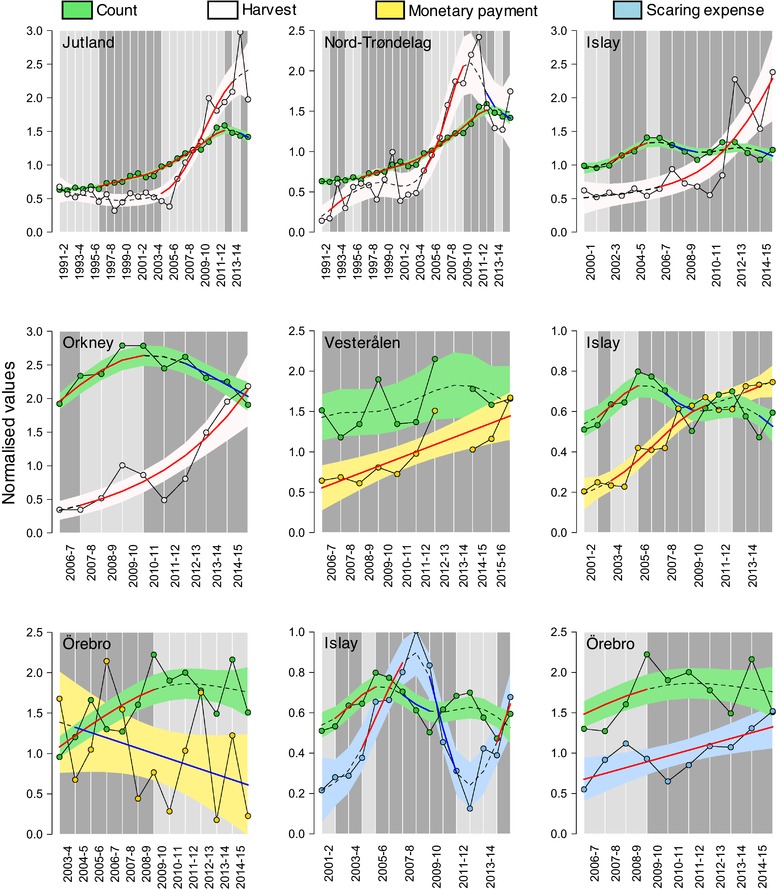
Long‐term synchrony in harvest, monetary payment, and scaring effort trends relative to population count trends for the different case studies considered. Trends were estimated using generalized additive models with a corrective AR(1) term for residual autocorrelation. Model‐fitted values were normalized by dividing estimates by the mean of the entire corresponding time series. Joined points represent observed values over time. Dashed lines denote fitted trends, with full red and blue sections representing periods of significant upward or downward trend, respectively. Light and dark gray backgrounds reflect time steps for which estimated trends in counts and management effort were synchronous and asynchronous, respectively

Estimated trends in reported hunting bag from Jutland in Denmark showed significantly higher synchrony with pink‐footed goose counts than expected by chance (Table 2, Figure 3). Despite this, the rate of change during the period of simultaneous significant increase observed between 2004 and 2012 was much higher for hunting bags (mean % change of 17.5) than for counts (mean % change of 6.2). In contrast, trends in reported hunting bag for pink‐footed geese from Nord‐Trøndelag in central Norway and for barnacle geese on Islay were random and significantly asynchronous, respectively, relative to population count trends (Figure 3). In Orkney, trend changes in the number of licenses granted and greylag goose counts did not show significant synchrony or asynchrony over time (Table 2, Figure 3).

**Table 2:** Long‐term synchrony between management effort and population count trends. Significance was assessed using binomial tests based on the observed and expected probability of synchronized changes

Strategy	Case study	Management effort trend	No. of time steps	No. of synchronized changes^a^	Observed probability^b^	Expected probability^c^	*P*‐value
Harvest	Jutland	Reported winter hunting bag	24	14	0.58	0.29	< 0.01
	Nord‐Trøndelag	Reported winter hunting bag	24	9	0.38	0.29	0.37
	Islay	Reported winter hunting bag	15	3	0.2	0.6	< 0.01
	Orkney	Total number of licenses	9	3	0.33	0.22	0.43
Monetary payment	Vesterålen	Average subsidy per application	10	0	0	1	<0.001
	Islay	Average subsidy per participant	14	7	0.5	0.64	0.28
	Örebro	Average compensation per report	12	6	0.5	0.5	1
Scaring	Islay	Total scaring expenses	14	4	0.29	0.64	< 0.01
	Örebro	Total scaring subsidy	9	3	0.33	0.67	0.07

a The number of contemporaneous trend step changes showing the same behavior.

b The proportion of times steps showing synchronized behavior.

c The proportion of time steps showing neither a significant increasing nor decreasing count trend.

Estimated trends in average monetary payments were either random, in the case of Islay and Vesterålen, or significantly asynchronous, in the case of Örebro, relative to simultaneous count trends for pink‐footed geese and cranes, respectively (Table 2, Figure 3). Average subsidy per application in Vesterålen increased significantly over time (GLM: 184.1 ± 44.9 euros increase per year on average, *P* < 0.01), matching an upward but nonsignificant trend in the combined counts for Svalbard pink‐footed and barnacle geese. Compensation payments and scaring subsidies associated with the management of common cranes in the Örebro county of Sweden showed nonsignificant negative (GLM: ‐96.4 ± 68.8 euros per year, *P* = 0.189) and significant positive (GLM: 4,069.7 ± 1,187.0 euros, *P* < 0.01; Figure 3) trends over time, respectively, which were concurrent with an initially increasing and then stable population count trend. The estimated trend in scaring expenses on Islay was significantly asynchronous relative to that of goose counts (Table 2).

### 3.2 Short‐term synchrony

**Figure 4:**
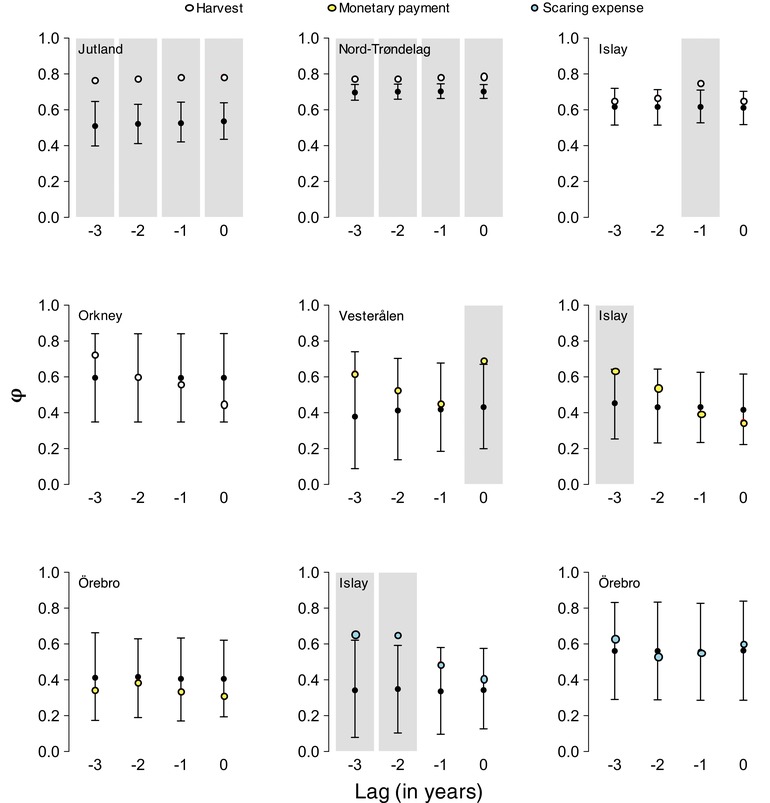
Observed and expected measures of short‐term synchrony (φ) between management effort and population counts for time lags of 0 to 3 years. Black dots with 95% CI brackets represent expected distributions obtained by randomizing the time series. Light gray shading denotes cases of significant synchrony

Annual changes in counts and reported hunting bags were found to be synchronized more than expected by chance in the case of pink‐footed geese in Jutland and Nord‐Trøndelag, but not for barnacle geese on Islay (Figure 4). Rather, for the latter, the number of geese shot during the current winter was more related to the number of geese counted the previous winter (φ_obs_ = 0.751, φ_exp_ = 0.617, *P* < 0.05). It is important to note that, due to strong serial autocorrelation in the time series of counts and hunting bags for both Jutland and Nord‐Trøndelag, synchrony remained high regardless of the time lag considered (see Supporting Information S5). In both cases, however, it was at its highest when no time lag was present. Although no significant synchrony or asynchrony was detected in Orkney, there was a tendency for counts and number of licenses issued to become less asynchronous with increasing time lag.

Changes in average subsidy per application in Vesterålen were more strongly synchronized with changes in the average number of geese recorded the same year (φ_obs_ = 0.690, φ_exp_ = 0.428, *P* < 0.05) than the previous year (φ_obs_ = 0.453, φ_exp_ = 0.423, *P* = 0.395; Figure 4). Compensation payments on Islay and in Örebro tended to be more asynchronous with contemporaneous counts than with lagged counts. In particular, average compensation payment per scheme participant on Islay was significantly more synchronized with the count from 3 years prior (φ_obs_ = 0.633, φ_exp_ = 0.451, *P* < 0.05) than with any other count. Similarly, total expenses for scaring activities on Islay showed significant lags of 2 (φ_obs_ = 0.650, φ_exp_ = 0.349, *P* < 0.01) and 3 (φ_obs_ = 0.655, φ_exp_ = 0.345, *P* < 0.01) years with the combined winter counts of white‐fronted and barnacle geese. Lastly, changes in scaring subsidies in Örebro showed no evidence of short‐term synchrony or asynchrony with common crane counts (φ_obs_ = 0.599, φ_exp_ = 0.562, *P* = 0.381 for a time lag of 0; Figure 4).

## 4 DISCUSSION

Our study reveals inconsistent patterns of synchrony and asynchrony between changes in impact management effort and population counts of large grazing bird species across case studies in northern Europe. In highlighting this variation, we provide valuable insights into potential indicators of social conflicts involving wildlife conservation and agricultural activities. Indeed, perceived randomness and inconsistent changes in management effort relative to wildlife impacts in the short and long terms have been shown to influence the level of trust stakeholders place on the decision‐making process (Young et al., 2016), as well as their overall responsiveness to policy change (Olson et al., 2015). Overall, our study highlights a need for more timely responses of management to changes in species counts so as not to unexpectedly influence social conflicts resulting from wildlife impacts (Redpath et al., 2013, Tuvendal & Elmberg, 2015; Young et al., 2010). Although we focus on large grazing bird populations, we expect our findings to be relevant to the management of conflicts involving other taxa, often in similar areas (e.g., wild boar *Sus scrofa*; Massei et al., 2015).

Long‐term patterns of synchrony and asynchrony in hunting bags need to be considered within the context of specific management aims, which may vary across space and time (Madsen et al., 2017; McKenzie & Shaw, 2017). Asynchrony can be expected if the aim is to promote growth of a vulnerable population, such as the Svalbard pink‐footed goose for which the initial significant increase in population count was accompanied by a stable hunting bag in Jutland prior to 2010 (Madsen, Christensen, Balsby, & Tombre, 2015). However, asynchrony can also occur when the aim is to purposefully reduce or stabilize an overabundant population (Menu, Gauthier, & Reed, 2002). This has been the case in more recent years for all the harvest examples considered here. Regardless of whether management is expected to result in synchrony or asynchrony, we emphasize the importance of closely monitoring and adapting harvest levels to ensure populations do not go above or below stated targets (Fryxell et al., 2010; Madsen et al., 2017).

Similarly, a time lag in management actions is to some degree inevitable and depends on the specific management process at hand. Annual hunting bags in Jutland and Nord‐Trøndelag are based on population estimates obtained the previous year and complemented with an estimation of harvest‐induced mortality as well as a forecast of the breeding output (Madsen et al., 2017). On Islay and in Orkney, hunting bags are largely based on the previous winter population count, while farm compensation payment rates for a given year on Islay relate to goose counts averaged over the past 5 to 7 years (McKenzie & Shaw, 2017). Although longer time delays are known to affect wildlife population dynamics (Fryxell et al., 2010), managers may require considerable time for data to be gathered at larger spatial scales in order to adjust hunting bags for migrating species, or wait for periodic reviews of government funding before altering monetary payment rates (Nyhus et al., 2005). Nevertheless, managers should strive to include time lags into adaptive management strategies, as has been done along the Svalbard pink‐footed goose flyway.

One surprising result is the absence of time lag in the synchrony between subsidies and population counts in Vesterålen, despite subsidies being allocated before geese arrive in the spring and based on previous counts (Tombre, Eythórsson, & Madsen, 2013). One explanation for this could be that managers benefit from information obtained at other flyway sites during the winter and are able to better predict numbers reaching Vesterålen during the spring (Tombre, Madsen, & Bakken, 2010). In other parts of Norway, such as in Nord‐Trøndelag, subsidies are based on regularly updated species distribution models (Madsen, Bjerrum, & Tombre, 2014). Although Eythórsson, Tombre, and Madsen (2017) highlight a positive relationship between goose numbers and total subsidy budget in Nord‐Trøndelag, they caution that political pressures may still cause asynchrony. This highlights the importance of scientific monitoring and research in the implementation of management schemes, but also clear communication of findings to managers and stakeholders (Tombre et al., 2013). Models in particular are powerful tools to support decision‐making in complex systems that are affected by uncertainty (Bunnefeld, Nicholson, & Milner‐Gulland, 2017), as demonstrated in the management of large grazing birds in North America (Menu et al., 2002).

Lack of short‐term synchrony can be seen as both a cause and a consequence of social conflict between parties invested in agricultural activities and wildlife conservation. Observed inconsistencies may arise as a result of competing interests influencing the decision‐making process. This may result in compromises that, although conducive to the attenuation of social conflicts (Liukkonen, Mykrä, Bisi, & Kurki, 2009), lead to suboptimal changes in management. Conflicts may also arise from inconsistent changes in management effort relative to the perception of how a population and the resulting agricultural damages are changing in the short term (Eriksson, Sandström, & Ericsson, 2015). On Islay and in Örebro, for instance, average monetary payments tended to decrease when observed goose and crane numbers increased, respectively. However, this tended to occur at the same time as rapid increases in culling quotas on Islay and the employment of full‐time scarers in Örebro, reflecting shifts toward more proactive solutions (see Supporting Information S6; Nyhus et al., 2005).

The limitations of our study open up important avenues for future research. First, we make the assumption that the relationship between population count and agricultural damage is linear. However, quantitative studies linking large grazing bird numbers to incurred damage remain scarce (Fox et al., 2017). Second, although our approach accounted for uncertainty in the estimation of population trends, we were unable to consider measurement error associated with individual counts. We recommend accounting for potential bias in the observation process when setting policy, for instance, by using a management strategy evaluation framework (Bunnefeld et al., 2011). Third, the indicators used to track changes in management effort may not be fully representative of the actual actions undertaken on the ground. For instance, they do not consider spatial variation in scaring and compensation levels, nor the fact that hunting bags may be liable to misreporting (Christensen, Madsen, Asferg, Hounisen, & Haugaard, 2017). However, such information is rarely recorded consistently and in a manner that would enable more widespread analysis of management time series (Tombre et al., 2013). Last, the existence of synchrony versus asynchrony may also be dependent on the spatial scale at which the population indicator is measured. For instance, national monitoring centers collate information simultaneously across the pink‐footed goose flyway to establish a total count, which is then used to apportion hunting bag quotas across relevant flyway zones (Madsen et al., 2017). Thus, it is reasonable to expect that synchrony will increase as the spatial scales used to measure management and population size become more comparable, but more research is needed to quantify this.

Conflicts between wildlife conservation and agricultural activities are becoming more widespread, threatening biodiversity and food security globally. As demonstrated in this study, the analysis of historical time series provides a useful evaluation of conflict management. Despite this, quantitative data on the history of management actions remain scarce and we urge managers and researchers to better document actions taken to promote transparency and evaluation. We also emphasize the importance of using models to support management decisions. A structured approach to decision‐making paired with adaptive co‐management could allow for quicker cycles of up to date population data, their subsequent translation into appropriate management actions, and the monitoring of their implementation by people on the ground. Socio‐ecological studies are required to clarify the relationship between changes in management actions and changes in stakeholder attitudes and behavior, thus enabling a sustainable process of conflict mitigation to be reached.

## Supporting information


**S1** Details relating to time series of management effort

**S2** Population and management effort time series

**S3** GAM implementation and trend estimation

**S4** Estimation of short‐term synchrony

**S5** Autocorrelation coefficients for flyway counts of Svalbard pink‐footed geese and hunting bags in Jutland and Nord‐Trøndelag

**S6** Estimated trend in the ratio of monetary payments to scaring expenses for both the Islay and Örebro case studies
