# Supplementary material for: Time series analysis reveals synchrony and asynchrony between conflict management effort and increasing large grazing bird populations in northern Europe
Source: Conserv Lett. 2018 Mar 25;12(1):e12450. doi: 10.1111/conl.12450 (PMC6472567; doi:10.1111/conl.12450)
Supplement: Supplementary file 1 — S1 Details relating to time series of management effort [file CONL-12-na-s001.pdf]

SUPPORTING INFORMATION S1 – Details relating to time series of management effort

Authors: Cusack et al.

We collected annual time series of management effort aimed at mitigating the wildlife impacts of each target species. Harvesting effort, including open season harvesting, lethal scaring and culling, was represented by either the reported winter hunting bag for the target species (see McKenzie & Shaw, 2017 for details on the Islay goose harvesting scheme; Madsen et al., 2015 and Clausen et al., 2017 for hunting bag reporting and estimation in Jutland and Nord-Trøndelag) or the total number of issued shooting licences (see Churchill & Skene, 2016 for the case of Orkney). Because reported hunting bags for Orkney were only available from 2012, we used the number of licences granted to shoot both resident and migratory greylag geese between 2006 and 2015 as an indicator of harvesting effort (Churchill & Skene, 2016). In the case of Islay, harvest included animals shot under special derogation (European Commission, 2009) as part of lethal scaring and culling implemented before and after the commencement of the management scheme in 2014 (McKenzie & Shaw, 2017), respectively. Although we recognise that open season hunting, lethal scaring and culling differ in their aim and number of birds shot (Månsson 2017), here we consider them as part of the same harvesting spectrum.

Time series of monetary payments relating to crop damage were collated for Islay (subsidy payment per scheme participant, see McKenzie & Shaw, 2017), Örebro (compensation payment per damage report, see Nilsson, 2016), and Vesterålen (average subsidy amount per application, see Eythórsson et al., 2017). We emphasise that monetary payments on Islay and in Vesterålen represent subsidies as not based on verified damage to crops, but rather on goose numbers counted in previous years. In contrast, compensation given to farmers in Örebro followed inspection of crop damage by local authorities. Scaring effort in the case of Islay was represented by total expenses allocated to scaring activities, which

included the employment of a goose scarer who managed a range of scaring devices, and contracted marksmen who fulfilled the quota set for lethal scaring purposes (McKenzie, 2014). In the case of Örebro, scaring expenses related to the employment of a scarer and purchase of associated scaring equipment (e.g. propane canons, kites and rotating mirrors; Nilsson et al., 2016). All nominal monetary values were adjusted for inflation using the percentage change (or annual growth rate) in the consumer price index (CPI) of the corresponding country (as reported by OECD, 2017). Importantly, harvesting, monetary payments and scaring activities can be implemented simultaneously and are thus not necessarily mutually exclusive activities at a given point in time.

References

Churchill, G., & Skene, C. (2016). *Orkney resident greylag goose adaptive management pilot 2012 to 2017: Annual report for 2015 season*. Scottish National Heritage.

Clausen, K. K., Christensen, T. K., Gundersen, O. M., & Madsen, J. (2017). Impact of hunting along the migration corridor of pink-footed geese *Anser brachyrhynchus* – implications for sustainable harvest management. *Journal of Applied Ecology*. DOI:10.1111/1365-2664.12850

Eythórsson, E., Tombre, I. M., & Madsen, J. (2017). Goose management schemes to resolve conflicts with agriculture: theory, practice and effects. *Ambio, 46*, 231–240. DOI:10.1007/s13280-016-0884-4

Madsen, J., Christensen, T. K., Balsby, T. J., & Tombre, I. M. (2015). Could have gone wrong: effects of abrupt changes in migratory behaviour on harvest in a waterbird population. *PLoS ONE, 10*, e0135100. DOI:10.1371/journal.pone.0135100

Månsson, J. (2017). Lethal scaring–Behavioral and short-term numerical response of greylag goose *Anser anser*. *Crop Protection, 96*, 258–264. DOI:10.1016/j.cropro.2017.03.001

McKenzie, R. (2014). *Islay Sustainable Goose Management Strategy October 2014 – April 2024*. Scottish Natural Heritage.

McKenzie, R., & Shaw, J. M. (2017). Reconciling competing values placed upon goose populations: The evolution of and experiences from the Islay Sustainable Goose Management Strategy. *Ambio, 46*, 198–209. DOI:10.1007/s13280-016-0880-8

Nilsson, L. (2016). *Common crabs in agricultural landscapes: linking space use and foraging patterns to conservation and damage prevention.* Thesis. Acta Universitatis agriculturae Sueciae, Swedish University of Agricultural Sciences.

Nilsson, L., Bunnefeld, N., Persson, J., & Månsson, J. (2016). Large grazing birds and agriculture—predicting field use of common cranes and implications for crop damage prevention. *Agriculture, Ecosystems & Environment, 219*, 163–170. DOI:10.1016/j.agee.2015.12.021

OECD (2017). Inflation (CPI) (indicator). DOI:10.1787/eee82e6e-en (Accessed on 29 August 2017)
