# Supplementary material for: Time series analysis reveals synchrony and asynchrony between conflict management effort and increasing large grazing bird populations in northern Europe
Source: Conserv Lett. 2018 Mar 25;12(1):e12450. doi: 10.1111/conl.12450 (PMC6472567; doi:10.1111/conl.12450)
Supplement: Supplementary file 3 — S3 GAM implementation and trend estimation [file CONL-12-na-s003.pdf]

SUPPORTING INFORMATION S3 – GAM implementation and trend estimation

Authors: Cusack et al.

For each case study, we modelled separate trends for counts and management effort over time using Generalised Additive Models (GAMs; Wood, 2004). In both cases, we accounted for first-order auto-regressive processes by including a corrective term for residual auto-correlation (i.e. AR(1)). Preliminary analyses revealed that GAMs fitted to time series of less than 10 consecutive years produced either linear or over-fitted trends. In these cases, we assumed a linear trend over time and fitted Generalised Linear Models (GLMs). We set the model error distribution to quasi-Poisson for count and hunting bag response variables to account for over dispersed count data, and to Gaussian for response variables relating to monetary payments and scaring expenses.

To test whether GAM trends in species counts and management effort were synchronised, we identified periods of significantly increasing or decreasing trends by producing 10 000 variants of each fitted trend and creating a distribution of fitted values at each time point. Fitted trend variants were created by sampling from a multivariate normal distribution parameterised using the fitted model coefficients and the variance-covariance matrix of the coefficients (Knape, 2016). For each time point, the probability of the trend being significantly increasing or decreasing was calculated as $P = k/(K+1)$ where k is the number of positive or negative simulated changes, respectively, and K is the total number of simulations. We applied a sequential Bonferroni correction to P to account for multiple significance tests.

References

Knape, J. (2016). Decomposing trends in Swedish bird populations using generalized additive mixed models. Journal of Applied Ecology, 53, 1852–1861. DOI:10.1111/1365-2664.12720

Wood, S. N. (2004). Stable and efficient multiple smoothing parameter estimation for generalized additive models. Journal of the American Statistical Association, 99, 673–686. DOI:10.1198/016214504000000980
