# Supplementary material for: Time series analysis reveals synchrony and asynchrony between conflict management effort and increasing large grazing bird populations in northern Europe
Source: Conserv Lett. 2018 Mar 25;12(1):e12450. doi: 10.1111/conl.12450 (PMC6472567; doi:10.1111/conl.12450)
Supplement: Supplementary file 4 — S4 Estimation of short‐term synchrony [file CONL-12-na-s004.pdf]

SUPPORTING INFORMATION S4 – Estimation of short-term synchrony

Authors: Cusack et al.

We applied a measure of synchrony combined with a Monte Carlo randomisation procedure to test whether annual changes in count and management effort were more synchronous than expected by chance in the short term. Loreau & de Mazancourt (2008) introduced a measure of the strength of synchrony between multiple species within a given ecological community, which is defined as:

$$\varphi = \sigma^2 \left[\sum_{i=1}^{N} x_i(t)\right] / \left(\sum_{i=1}^{N} \sigma[x_i(t)]\right)^2$$

in which the numerator represents the community temporal variance (x_i is the abundance time series of species *i* and *N* is the total number of species) and the denominator represents the sum of the population-level standard deviations squared (see Loreau & de Mazancourt, 2008; Gouhier & Guichard, 2014). As a result, φ is standardised between 0 (perfect asynchrony) and 1 (perfect synchrony). Rather than consider multiple species, we consider only two time series, one for population count and the other for management effort. Thus, in our case, community-wide variance refers to the variance associated with the sum of both count and management effort time series.

References

Gouhier, T. C., & Guichard, F. (2014). Synchrony: quantifying variability in space and time. *Methods in Ecology and Evolution, 5*, 524–533. DOI:10.1111/2041-210X.12188

Loreau, M., & de Mazancourt, C. (2008). Species synchrony and its drivers: neutral and nonneutral community dynamics in fluctuating environments. *The American Naturalist, 172*, 48–66. DOI:10.1086/589746
