# Supplementary material for: Time series analysis reveals synchrony and asynchrony between conflict management effort and increasing large grazing bird populations in northern Europe
Source: Conserv Lett. 2018 Mar 25;12(1):e12450. doi: 10.1111/conl.12450 (PMC6472567; doi:10.1111/conl.12450)
Supplement: Supplementary file 5 — S5 Autocorrelation coefficients for flyway counts of Svalbard pink‐footed geese and hunting bags in Jutland and Nord‐Trøndelag [file CONL-12-na-s005.pdf]

**SUPPORTING INFORMATION S6**

**Authors:** Cusack et al.

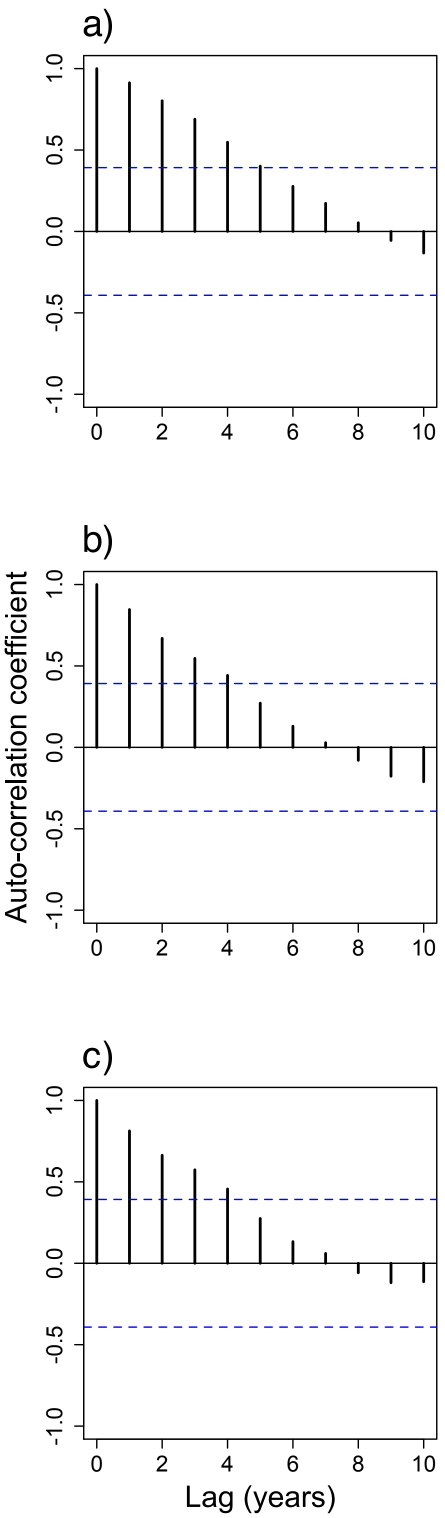


Auto-correlation of time series of pink-footed goose (a), reported winter hunting bag in Jutland (b) and Nord-Trøndelag (c) for lags of one to 10 years. Dashed blue lines represent thresholds for significance.
