# Supplementary material for: Time series analysis reveals synchrony and asynchrony between conflict management effort and increasing large grazing bird populations in northern Europe
Source: Conserv Lett. 2018 Mar 25;12(1):e12450. doi: 10.1111/conl.12450 (PMC6472567; doi:10.1111/conl.12450)
Supplement: Supplementary file 6 — S6 Estimated trend in the ratio of monetary payments to scaring expenses for both the Islay and Örebro case studies [file CONL-12-na-s006.pdf]

SUPPORTING INFORMATION S6

Authors: Cusack et al.

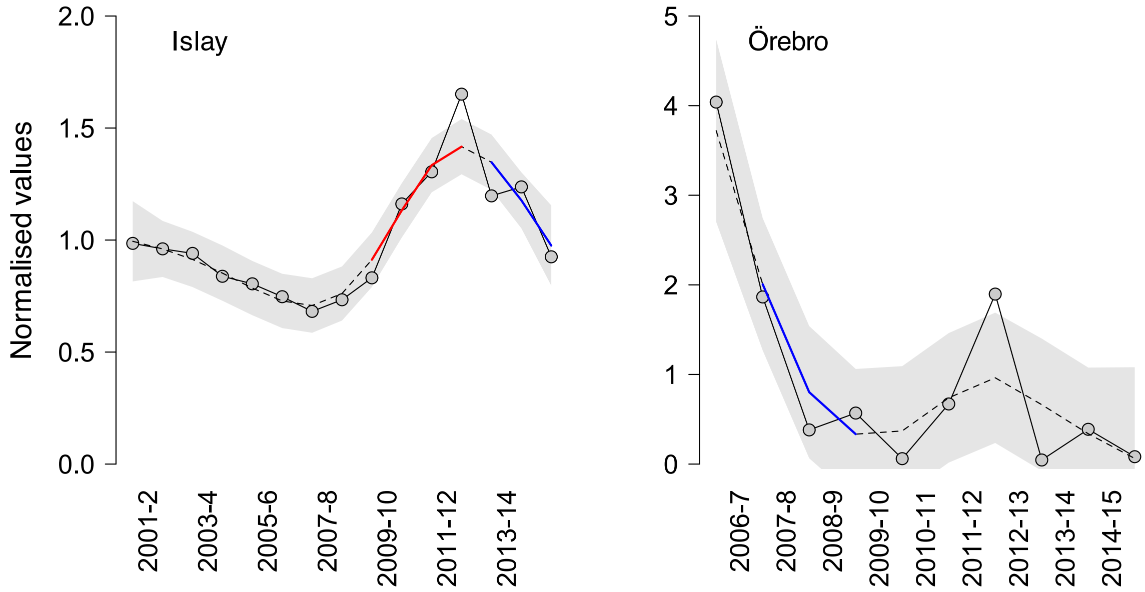

We estimated the trend in the ratio of total budget allocated to monetary payments to total budget allocated to scaring expenses for Islay and Örebro in order to capture relative changes in the financing of both activities over time. Trends were estimated using Generalised Additive Models with a corrective AR(1) term for residual auto-correlation. Joined points in the above figure represent observed values over time. Dashed lines denote fitted trends, with full red and blue sections representing periods of significant upward or downward trend, respectively. This analysis revealed contrasting patterns between Islay and Örebro. On Islay, the budget allocated to monetary expenses increased relative to that for scaring expenses between 2009 and 2012, but decreased thereafter. In contrast, in Örebro the ratio of monetary payment to scaring expense budgets declined significantly between 2007 and 2009. The ratio then remained stable from 2010 to 2015.
